# Modulation of rabbit corneal epithelial cells fate using embryonic stem cell extract

**Published:** 2010-06-23

**Authors:** Weijiao Zhan, Zhiping Liu, Ying Liu, Qicheng Ke, Yuanyuan Ding, Xiaoyan Lu, Zhichong Wang

**Affiliations:** 1 State Key Laboratory of Ophthalmology, Zhongshan Ophthalmic Center, Sun Yat-sen University, Guangzhou, Guangdong, China; 2 Center for Stem cell Biology and Tissue Engineering, Sun Yat-sen University, Guangzhou, Guangdong, China

## Abstract

**Purpose:**

To develop a new culture system to cultivate differentiated autologous cells in vitro for cell therapy and tissue engineering.

**Methods:**

After incubation in murine embryonic stem cell (ESC) extract for 1 h, streptolysin-O (SLO) permeabilized cells were resealed with CaCl_2_ and continually cultured for weeks. The morphological study was analyzed by light microscopy. Isolated colonies were selected and expanded to establish cell lines. Octamer-4 (Oct-4), stage-specific embryonic antigen-1 (SSEA-1), transformation-related protein 63 (p63), ATP-binding cassette subfamily G, member 2 (ABCG2), and cytokeratin3 (K3) were detected by indirect immunofluorescent staining. *Oct-4*, *K3*, and *p63* were also detected by RT–PCR analysis. To examine the stemness characteristics of the induced cells, both alkaline phosphatase (AKP) staining and tumorigenicity detection were performed, respectively.

**Results:**

Reprogramming was induced in corneal epithelial cells. The reprogrammed cells showed characteristics similar to ESCs in the early weeks, including colony formation, positive AKP staining, and multi-potential differentiation in vivo. Oct-4 and SSEA1 protein expression was upregulated. However, these changes were not persistent or stable. With the passage of time, the colonies became flat. The ESC markers were downregulated, while epithelial cell related proteins gradually increased.

**Conclusions:**

Less terminal differentiated rabbit corneal epithelial cells could be induced to a more pluripotent state with embryonic stem cell extract (ESC-E). These cells have the potential to return to the beginning of their own lineage and obtain the ability of long-term growth. Our ﬁndings indicate that this culture system can generate low-immunogenic autologous cells for use in regenerative medicine.

## Introduction

Corneal damage and limbal stem cell deficiency may lead to conjunctivalization of the cornea and subsequent loss of vision. Stem cells undergo self-renewing division and can give rise to more committed progenitor cells that can differentiate into a variety of tissues. The discovery of limbal stem cells provides ideal biologic material for corneal diseases. However, the adult limbal stem cells from patients are difficult to isolate and expand in a timely manner. Dedifferentiation or reprogramming of adult somatic cells into a multipotent state may provide an attractive source of patient-specific stem cells for regenerative medicine [1]. In our previous study [2], we explored embryonic stem cell (ESC) conditioned medium (ESC-CM), which had the protective capacity in promoting survival and proliferation of the corneal epithelial cells from rabbit peripheral corneal tissue. We also found these cells were ESC-CM dependent. After removing the ESC-CM, the cells lost their long-term proliferative capacity.

SCNT (somatic cell nuclear transplantation) suggests that the oocyte environment provides all the factors necessary for turning differentiated nuclei into pluripotent nuclei, although the efficiency of the process is low. Recently, several studies demonstrated that exposure of somatic cell nuclei to ESC-derived cell-free factors/proteins could drive somatic cell reprogramming [1,3-5], which proved that the multipotent epigenome could be activated in somatic cells without nuclear transfer or expression of defined genes.

Indeed, alterations in the fate of one type of differentiated somatic cell by cell-free extracts from another, leading to the acquisition of donor cell characteristics and functions by recipient cells, have been previously reported [6-8].

In the present study, we report that streptolysin-O (SLO) -permeabilized primary rabbit corneal epithelial cells were markedly reprogrammed after exposure to ESC-E (murine embryonic stem cell extract). We demonstrated the induction of reactivation of ES-cell-specific gene expression (Octamer-4 [*Oct-4*] and stage-specific embryonic antigen-1 [*SSEA-1*]). These induced cells had ES-cell-like morphology and growth properties, as well as multilineage differentiation potential in vivo. However, in later weeks, the reprogrammed cells took on some properties of epithelial cells. This proved that less terminally differentiated rabbit corneal epithelial cells could be induced to a more pluripotent state with embryonic stem cell extract (ESC-E). Nevertheless, these cells have the potential to return to the beginning of their own lineage and obtain the ability of long-term growth. We attempted to develop a new culture system using ESC-E to cultivate the differentiated autologous cells in vitro for cell therapy and tissue engineering.

## Methods

### Animals and cells

Young New England white rabbits (aged 10 weeks and weighed 2–3 kg) and Balb/c nude mice (aged 2–3 weeks) were purchased from the Animal Laboratory of Sun Yat-sen University (Guangzhou, China) and Southern Medical University (Guangzhou, China), respectively. All animal experiments were performed with permission of the Medicine Ethics Committee in Zhongshan Ophthalmic Center, Sun Yat-sen University, and complied with Association for Research in Vision and Ophthalmology (ARVO) statement of the use of animals in ophthalmic and visual research. Mouse embryonic stem cell line E14 (ES-E14) cells were generously provided by Professor P. Xiang in Sun Yat-sen University, China.

Mouse ES-E_14_ cells were plated at a density of 400/cm^2^ on 1% gelatin (Sigma, St. Louis, MO)–coated tissue culture dishes containing mouse ESC culture medium [9] with 50% of the medium changed every day. ESCs were stained with anti-mouse Oct-4 antibody (Chemicon, Temecula, CA) to determine the undifferentiated status of ESCs before and after collection.

Primary corneal epithelial cells were cultured from rabbit peripheral corneal tissue explants (a ring between 2 mm anterior to the corneoscleral junction and 3 mm posterior to the corneal central point) in corneal epithelium medium on tissue culture dishes (BD Falcon/BD Biosciences, Franklin Lakes, NJ) [10]. After the cells reached 60%~80% confluence (7~10 days), the epithelial cells were digested and counted.

### Cell extract preparation

Cell extracts were prepared following the procedures described by Taranger et al. [4]. Briefly, to prepare murine ES (mES) cell or rabbit paracentric corneal epithelial cell extracts, cells were washed twice in PBS and once in cell lysis buffer (20 mM HEPES pH 8.2, 50 mM NaCl, 5 mM MgCl_2_, 1 mM dithiothreitol, 0.1 mM phenylmethyl sulfonyl fluoride [PMSF], and 0.1% protease inhibitor cocktail; Sigma, St. Louis, MO), sedimented at 400× g, resuspended in 1.5 volume of cold cell lysis buffer and incubated for 40 min on ice. Cells were sonicated on ice using a sonicator (Ningbo Scientz Biotechnology CO, LTD, Ningbo, China) fitted with a 3 mm-diameter probe until all cells and nuclei were lysed, as judged by microscopy. The lysate was cleared at 15,000× g for 15 min at 4 °C. The supernatant was carefully collected and transferred into a new chilled 1.5 ml tube. To avoid ES cell contamination, the tube was snap-frozen in liquid N_2_ and stored at −80 °C for at least 2 weeks to remove living cells. About 5 µl of the extracts were used to determine protein concentration (approximately 15 mg/ml).

### SLO-permeabilization and cell extracts treatment

We optimized the Håkelien et al. [7] permeabilization protocol to avoid cell death for long-term suspension. Briefly, 5×10^5^ primary corneal epithelial cells were permeabilized with streptolysin-O (SLO) 0.25 μg (0.5 μg/ml) for 15 min at 37 °C with occasional agitation. After permeabilization, cells were resuspended in 150 µl cell extracts (mES cell extracts or control epithelial cell extracts) containing 1 mM ATP, 10 mM creatine phosphate, 25 μg/ml creatine kinase (Sigma), and 1 mM of each NTP, and incubated at 37 °C in an H_2_O bath with occasional agitation. For membrane resealing, cells were transferred into 5 ml corneal epithelial cell culture medium containing 2 mM CaCl_2_, seeded on a 25 cm^2^ culture flask, and cultured for 2 h in an incubator at 37 °C in a humidified atmosphere of 5% CO_2_. Then the Ca^2+^-containing medium was removed and replaced with complete corneal epithelial cell culture medium. The cells were placed back in the 5% CO_2_ incubator, the culture medium changed every other day, and subcultured at 1∶3 split ratio.

When colonies were found in P5 ESC-E-induced cells (ES cell extract treated paracentric epithelial cells, e-Pc), several colonies were picked up, transferred to the ES cell culture medium and expanded to establish cell lines. In vitro culture was associated with morphological observation, gene expression, and immunological analyses to evaluate induction of dedifferentiation. The following controls were set up and run in parallel to the reprogramming experiment: (a) black control, paracentric epithelial cells without any treatments (b-Pc) and (b) paracentric corneal epithelial cells treated with SLO and corneal epithelial cell extract (p-Pc). As b-Pc can be subcultured no more than 3–4 passages, p-Pc were transferred to the ES cell culture medium at passage 6 as control only.

### Histology and immunocytochemistry

After being cultured on 6-well slides for 24–48 h, cells were ﬁxed with 4% paraformaldehyde for 20 min and then processed using standard immunoﬂuorescence staining procedures. The primary antibodies were mouse anti-goat Oct-4 recognizing multiclonal Ab (mAb; Santa Cruz Biotechnology, Inc., Santa Cruz, CA), SSEA1 (Chemicon), K3/K12 mAb (Chemicon), p63 mAb (Chemicon), ABCG2 mAb (Santa Cruz Biotechnology, Inc.), and Vimentin mAb (Abcam, Cambridge, UK). Then, cells were incubated with Cy3 or FITC conjugated secondary goat anti-mouse IgG (Boster Biotech, Wuhan, China) for 1 h. Before being mounted, the nuclei were counterstained with Hoechst 33342. Then, the cells were observed with a confocal laser scanning microscope (LSM510META; CarlZeiss, Hamburg, Germany). Cells incubated with PBS instead of primary antibody were used as negative controls.

### Gene expression analysis

Total cellular mRNA was isolated from the cell cultures with Trizol Reagents (Invitrogen, Carlsbad, CA) according to the manufacturer’s instructions and was quantiﬁed by its absorption at 260 nm wavelength. The mRNA expression was detected by reverse transcriptase polymerase chain reaction (RT–PCR) with a housekeeping gene, glyceraldehyde-3- phosphatedehydrogenase (*GAPDH*), as an internal control. First-strand cDNA was synthesized from1 μg total RNA in 20 μl reaction mixture using a cDNA synthesis kit (MBI Fermentas, Burlington, Canada). PCR was then performed using speciﬁc primer pairs (Table 1) and PCR mix (Invitrogen, Carlsbad, CA) in a thermal cycle (Biotra, Goettingen, Germany).

**Table 1:** Primer sequences used for PCR.

**Gene**	**Sense primer**	**Anti-sense primer**	**Annealing temperature (° C)**	**PCR product (bp)**
*ΔNp63*	GAAAACAATGCCCAGACTCAATTT	TCTGCGCGTGGTCTGTGTTAT	54	127
*K3*	TCCGTCACAGGCACCAAC	TGCGTTTGTTGATTTCGTCT	54	175
*Oct-4*	CTTGGGCTACACTCAGGC	CTCAATACTCGTTCGCTTTC	60	238
*GAPDH*	GGAGCCAAAAGGGTCATC	CCAGTGAGTTTCCCGTTC	54	346

### Alkaline phosphatase (AKP) staining and teratoma formation examination

To visualize AKP activity, mES cell extract-induced cells were fixed and stained with the BCIP/NBT Phosphatase Substrate System (KLP, Gaithersburg, MD) following the manufacturer’s instructions and observed with a light microscope.

For teratoma formation examination, P9 (week 4) and P18 (week 8) e-Pc suspensions (2×10^7^/ml) and P2 b-Pc suspensions were collected and injected subcutaneously into six Balb/c nude mice, respectively. mES-E_14_ cells were used as positive controls and primary corneal epithelial cells were used as negative controls. After 80 days of cell injection, these nude mice were euthanized and examined for tumorigenicity.

## Results

### Cell morphology

**Figure 1:**
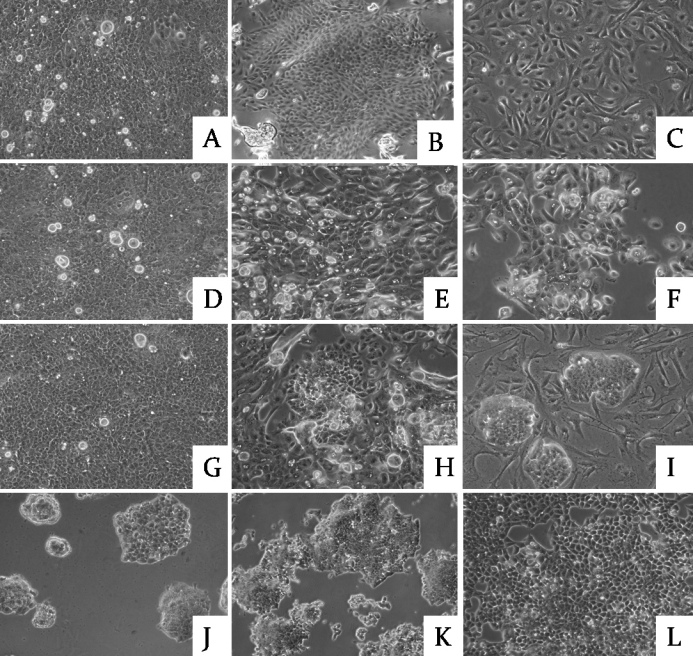
Representative phase-contrast micrographs of cells. **A**-**C**: SLO+ corneal epithelial cell extract-induced cells (p-Pc) P1 (day 8), P3 (day 12), P5 (day 14); **D**-**F**: cells without any treatment (b-Pc) P1 (day 3), P3 (day 9), P5 (day 14); **G**-**I**: SLO+ES cell extract-induced cells (e-Pc) P1 (day 8), P3 (day 12), P5 (day 14); **J**-**L**: e-Pc P9 (wk 4), P12(wk 6), P18 (wk 8). The magnification of panel **E** is 50×, the other panels are at 100×. The three groups maintained similar morphology at P1 (day 8; **A**, **D**, and **G**). Some fibroblast-like cells took place in p-Pc group (B and **C**). The P3 cells in b-Pc control exhibited aging appearance with vacuoles and larger size, and could not survive through P5 (**E** and **F**). At P3 (day 14) of e-Pc, among the aging epithelial cells, colonies with small cells and vague boundaries showed up (**H**). At P5 (week 3), colonies that closely resembled to mES colonies could be readily identified by their morphology (**I**). This phenomenon was maintained for at least 14 weeks in culture, corresponding to 26 passages, but the colonies gradually became flat (**J**-**L**).

Extract-induced cells, including p-Pc group and e-Pc group, took more time (6–8 days) to recover and reach confluence. They maintained morphology similar to the cells without any treatment (b-Pc) when the extract-induced cells reached confluence at P1 (day 8; Figure 1A,D,G). Some fibroblast-like cells took place in the p-Pc group (Figure 1B,C). The fibroblast-like cells could not survive through P6 and no colonies were found when they were transferred to mES culture medium. The P3 cells in b-Pc control exhibited an aging appearance with vacuoles and larger in size, partially detached from the dishes, and could not survive through P5 (Figure 1 E,F). At P3 (day 14) of the e-Pc, among the aging epithelial cells, colonies with small cells and vague boundaries showed up (Figure 1H). At P5 (week 3), colonies that closely resembled mES colonies could be readily identified by their morphology (Figure 1I). Then the colonies were picked up and expanded to establish cell lines in mES cell culture medium. This phenomenon was maintained for at least 14 weeks in culture, corresponding to 26 passages, but the colonies gradually became flat (Figure 1J-L).

### mRNA and protein expression proﬁles

**Figure 2:**
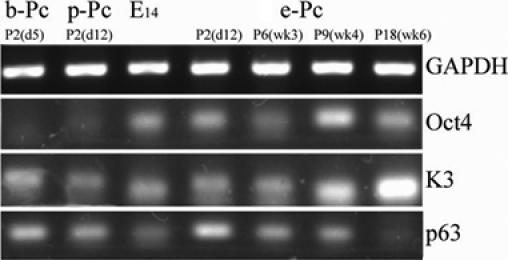
mRNA expression of *Oct-4*, *K3*, and *p63* with *GAPDH* as an internal control for P2 in all groups, E_14_ and P6, P9, P18 of e-Pc. After the mES cell extract treatment, *Oct-4* mRNA was detected in P2 (day 12), reached its peak at P9 (week 4), and decreased in later passages. It remained undetectable in the two control groups. Expression of corneal tissue-speciﬁc marker *K3* mRNA increased as passage in experiment group, and progenitor cell marker *p63* was also found in these cells.

**Figure 3:**
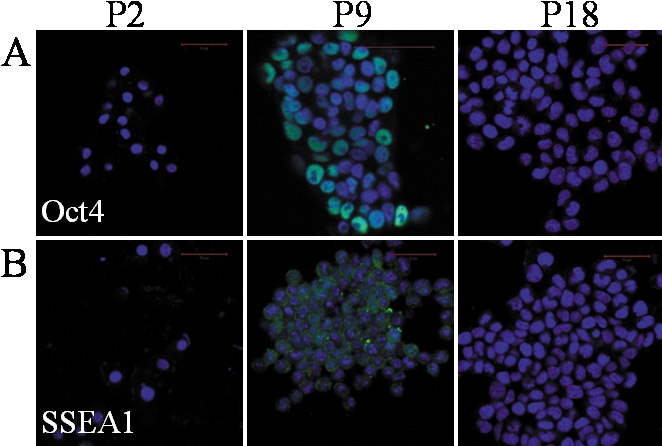
Expression of pluripotency-associated proteins Oct-4 and SSEA1 in e-Pc with immunoﬂuorescent staining. The scale bar represents 50 μm. Oct-4 and SSEA1 proteins were found in P9 (week 4), not in P18 (week 8) cells.

After the mES cell extract treatment, Oct-4 mRNA was detected in P2 (day 12), reached its peak at P9 (week 4), then decreased in later passages (Figure 2). The changes were confirmed by immunoﬂuorescent staining in P9 (week 4) and P18 (week 8; Figure 3A). Expression of SSEA1 protein was found in P9 (week 4), not in P18 (week 8; Figure 3B) with immunoﬂuorescent staining. These markers remained undetectable in the two control groups. This suggested that the ESC-E-induced cell lines were undergoing dedifferentiation.

**Figure 4:**
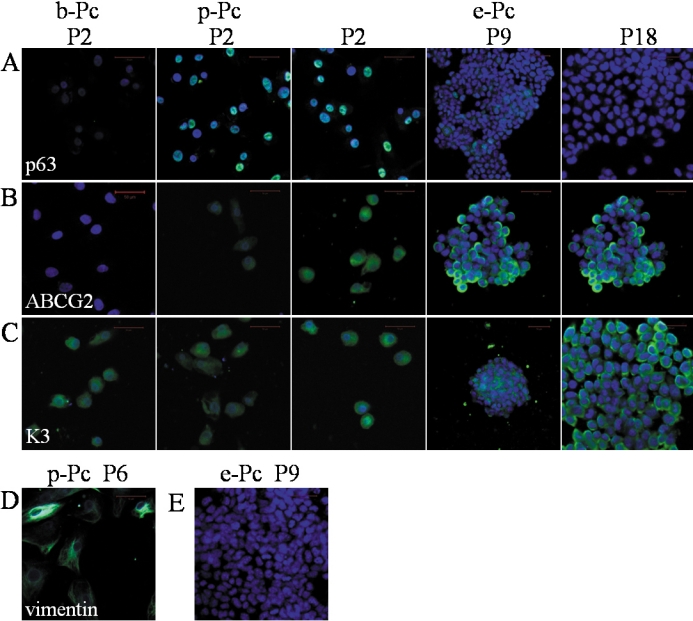
Expression of corneal-epithelium-related proteins in different conditions and passages with immunoﬂuorescent staining. **A**-**C**: p63, ABCG2, and K3 (green, positive cells; blue, nuclei) for P2 in all groups and P9, P18 of e-Pc. **D**: Vimentin for P6 in p-Pc group. **E**: Vimentin for P9 in e-Pc group. The scale bar in **B** represents 50 μm. Corneal tissue-speciﬁc marker K3 and progenitor cell markers, p63 or/and ABCG2 were still found in different passages of e-Pc. Vimentin, an intermediate ﬁlament protein and a characteristic of keratocytes and ﬁbroblasts was not detected in P9 cells of e-Pc. But it was positive in P6 of p-Pc.

We also detected the expression of corneal tissue-speciﬁc marker K3 [11] and the progenitor cell markers, p63 [12] or/and ABCG2 [13]. After colonies were selected, expression of *K3* mRNA increased as passage, and *p63* expression was also found in these cell lines (Figure 2). Immunoﬂuorescent staining confirmed the results (Figure 4). This suggested that full reprogramming to a pluripotent state had not been achieved, but the ESC-E-induced cells had the ability to return to the start of their lineage. Vimentin, an intermediate ﬁlament protein and a characteristic of keratocytes and ﬁbroblasts [14], was not detected in P9 cells of ESC-E group (Figure 3B), indicating that no ﬁbroblast contamination existed. In addition, we found that in P2, the expression of *p63* was more significant in e-Pc and p-Pc. The expression of vimentin was positive in P6 of p-Pc.

### Teratoma formation examination and alkaline phosphatase staining

**Figure 5:**
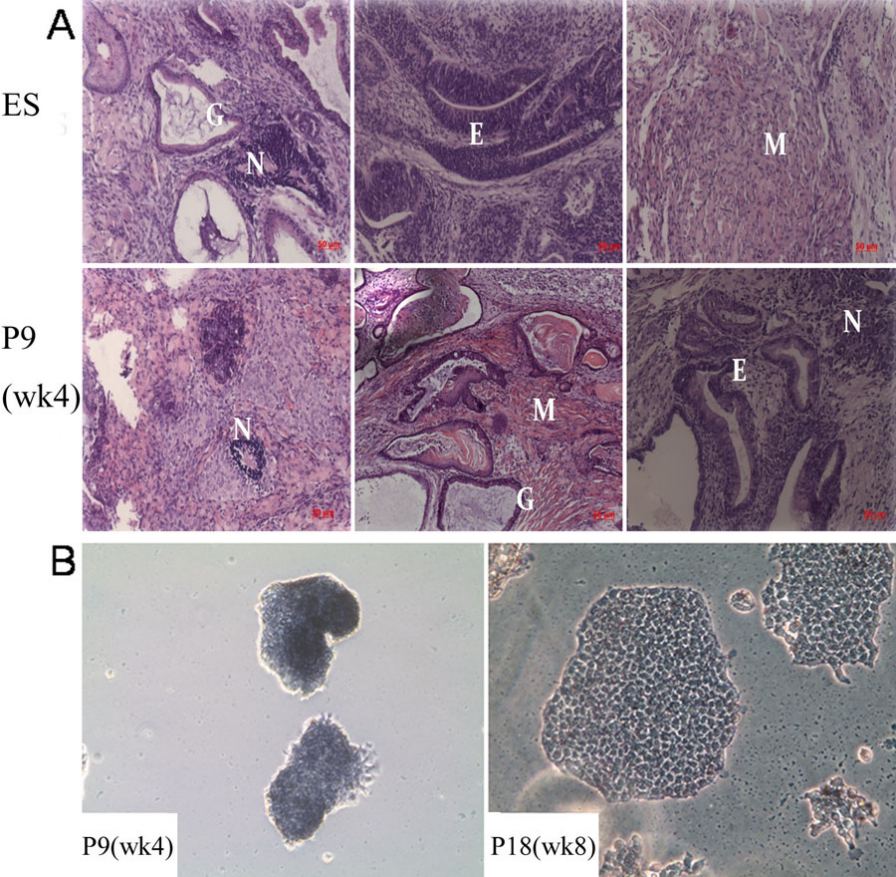
Teratoma formation examination and alkaline phosphatase (AKP) staining. **A**: HE staining shows teratoma from ES cells and P9 (wk 4) e-Pc containing multiple tissues, including epithelium (E), neural (N), muscle (M), and glandular structures (G). The scale bar represents 50 μm. **B**: AKP staining in P9 (wk 4) and P18 (wk 8) e-Pc (magnification, 100×). AKP staining was positive at P9 (week 4), and positive but weaker at P18 (week 8). Small and cohesive colonies were mostly observed in P9 cells. Flatter, larger and more migratory colonies were noted in P18 cells.

Human ES cells form teratomas when injected into immunocompromised mice [15], and has become a standard assay of pluripotency. Similarly, early passage of the ESC-E-induced cells (P9) and ES-E_14_ cells formed teratomas after subcutaneous injection into Balb/c nude mice. P18, CEC-E treated cells, and primary corneal epithelium injected cells did not form teratomas. Histological examination of the teratomas revealed that P9 ESC-E-induced cells were able to differentiate to three germ layers as ES-E_14_ cells, including the epithelium, neural epithelium, muscle, and various glandular structures (Figure 5A).

To perform AKP staining, P9 (week 4) and P18 (week 8) cells were seeded at a low cell density and cultured for a week. We found AKP staining was positive at P9 (week 4), and positive but weaker at P18 (week 8; Figure 5B). As far as colony morphology and size were concerned, small and cohesive colonies were mostly observed in cells of earlier passages; on the contrary, ﬂatter, larger, and more migratory colonies were noted in the later passages.

## Discussion

In the present study, we reported SLO-permeabilized primary rabbit corneal epithelial cells were markedly reprogrammed after exposure of mES cell extracts. After the cells were induced with mES cell extracts, we found that *Oct-4* mRNA was detectable at week 2; colonies closely resembling ES colonies were readily identified by their morphology at week 3 and stained positive for AKP. After colonies were picked up and expanded to establish cell lines in the ES cell culture medium, ES cell-specific protein expression (Oct-4 and SSEA1) was detected by immunoﬂuorescent staining. The cells took on a proliferative capacity and had teratoma formation capability after being injected into immunocompromised mice. However, in spite of these reprogramming changes, we also found that the colonies gradually became flat, and the expression of epithelium markers and ES cell-specific markers changed dramatically. This suggested that exposure to mES cell extracts had initiated a series of changes driving the somatic cells toward a more pluripotent state, but full reprogramming to a pluripotent state had not been achieved. The cells had the potential to dedifferentiate.

The studies of Qin et al. [16] and Håkelien et al. [7] indicated that the expression of new proteins was not a direct uptake from the extracts. Taranger et al. [4] demonstrated that the ES cell extracts induced a biphasic wave of *Oct-4* transcription and translation in 3T3 cells. The authors found that Oct-4 was detected as early as 1 h after recovery of the cells from the extracts, the level peaked at 24 h, then decreased and was barely detectable in the next 48 h. By 72 h, however, a second wave of Oct-4 was detected of amplitude similar to or higher than the first wave, and this wave persisted for at least 5 days. Oct-4 protein was also detected in these cells 5 and 10 weeks after extract treatment. The authors considered that the second elevation of Oct-4 in these cells was long-lasting. Taranger et al. [4] further concluded that whereas uptake of limited amounts of short-lived extract-derived Oct-4 protein probably occurred at the first wave, the two phases of *Oct-4* induction resulted from RNA Pol II-mediated transcriptional and translational activity in extract-induced cells. Considering no living cells and mRNA could survive through the long period of preparation and preservation of the extracts, the *Oct-4* mRNA we detected in P2 could not have come from the mES cell extracts. *Oct-4* mRNA, Oct-4 and SSEA1 proteins were expressed by the induced cells and located in the second wave. This indicated that the reprogramming of epithelial cells was successfully induced by mES cell extracts.

With subculturing, the colonies became flat, and the expression of *K3* increased. Progenitor cell markers p63 and ABCG2 were also detectable in cell lines. A previous study indicated that after exposure of 293T cells to mouse ES cell extracts, the expression levels of pluripotency genes continued to rise over the following 48 h, suggesting that long-term reprogramming of gene expression had occurred. However, some of the pluripotency genes were still significantly lower than that observed in the ES cells. This suggested that full reprogramming to a pluripotent state had not been achieved, but that exposure to ES cell extracts had initiated a series of changes driving the somatic cells toward a more pluripotent state [3]. Microarray analysis of gene expression in extract-induced 293T cells [4] indicated that the number of consistently upregulated genes decreased gradually from weeks 1 to 8, and the number of genes consistently downregulated gradually decreased, too. This suggested that the induced cells had the potential to dedifferentiate.

Accordingly, our observations demonstrated that mES cell extracts could reprogram somatic cells toward multipotency. The induced cells had the potential to dedifferentiate and obtained the capability for quick expansion and long-time subculture. The cells we obtained could be useful for corneal regenerative medicine. Of course, further experiments should be conducted, such as global expression and function analysis by comparing among rabbit ES cells, the reprogrammed cells, and corneal epithelial cells.
